# Supplementary material for: Analysis of secondary metabolite gene clusters and chitin biosynthesis pathways of Monascus purpureus with high production of pigment and citrinin based on whole-genome sequencing
Source: PLoS One. 2022 Jun 1;17(6):e0263905. doi: 10.1371/journal.pone.0263905 (PMC9159588; doi:10.1371/journal.pone.0263905)
Supplement: S1 Table — (DOCX) [file pone.0263905.s001.docx]

**S1 Table. The primers used in this study.**

| Primer name | Sequence (5′-3′) | Description |
| --- | --- | --- |
| RT-3524-F | AAGGGCCTCTTGACCTTTG | For RT-qPCR analysis of *MpigA* |
| RT-3524-R | CCTGAGACGAGTTCTTCGTATTT |  |
| RT-3525-F | GACCACGGTGCTGTATCTATG | For RT-qPCR analysis of *MPigR* |
| RT-3525-R | CTAAAGACCGCTGCTCATACTC |  |
| RT-3526-F | GAGGCCCTGATGGTTTAAGT | For RT-qPCR analysis of *MpigC* |
| RT-3526-R | CGTTTCTCGGAGGTGAGATAG |  |
| RT-3527-F | CGACCCGTCTGTCAAGTTTAT | For RT-qPCR analysis of *MpigD* |
| RT-3527-R | TCATCTTGCACAGGTCATCC |  |
| RT-3528-F | CAGACTTTCCATGGGAGACATC | For RT-qPCR analysis of *MpigE* |
| RT-3528-R | CTGCACTTCGGTCAGGTTATAC |  |
| RT-3529-F | TGGACCAGGTCGAGAAGTAT | For RT-qPCR analysis of *MpigF* |
| RT-3529-R | CGATCTTCTGGAGGCTGTATTT |  |
| RT-3530-F | CTGGAGGAGCATCGGAAAC | For RT-qPCR analysis of *MpigG* |
| RT-3530-R | CCACAACATCCTTCGTCTTGA |  |
| RT-3531-F | CCAGGGCCGCAAGTTTAT | For RT-qPCR analysis of *MpigH* |
| RT-3531-R | GATCCACGAGACGAAGAAGATG |  |
| RT-3532-F | CATCAGTGGACGCTGCATAA | For RT-qPCR analysis of *MpigI* |
| RT-3532-R | TCGTAGTGCCCAGGAAGAA |  |
| RT-3533-F | GAGAAGGAACAGCGTGGATTAC | For RT-qPCR analysis of *MpigJ* |
| RT-3533-R | CCGATCTTGGACTCAGGATAGA |  |
| RT-3534-F | AGGCTGATCTCTTCTCTCCTAC | For RT-qPCR analysis of *MpigK* |
| RT-3534-R | CCTGCTCAACTTGGTCTCTATC |  |
| RT-3536-F | GAGAAGACACCAGAGGAAGTTATT | For RT-qPCR analysis of *MpigM* |
| RT-3536-R | CTCAGAAAGACGACGGAAGAC |  |
| RT-3538-F | CGCAGAAACGCTCGACTATTA | For RT-qPCR analysis of *MpigP* |
| RT-3538-R | GTAGGGAGATGGCTGTAAACTG |  |
| RT-3539-F | CGCAGCTAGGCATTGTCATA | For RT-qPCR analysis of *MpigQ* |
| RT-5180-R | GCAAGCAAGAGCACAATCAA |  |
| RT-3836-F | GGTCATGTAGCTCTGGGATAAAG | For RT-qPCR analysis of *cit S* |
| RT-3836-R | CGGTATGTTCCAGGTTGAGATAG |  |
| RT-3837-F | GTGTCATCGACCACCTCTAAAT | For RT-qPCR analysis of *cit A* |
| RT-3837-R | CCTTGAACGGCTTCCTCTAAA |  |
| RT-3838-F | GAGCCATCACGCTTCTCTT | For RT-qPCR analysis of *cit B* |
| RT-3838-R | CGTCTTTGTTCGGAGGAACT |  |
| RT-3843-F | CACCATGTCAGCTGTGTCTTA | For RT-qPCR analysis of *cit C* |
| RT-3843-R | AGAGGCTCTGATGCTCTTCTA |  |
| RT-3840-F | GTAGATCACGCTGGAGTAGATG | For RT-qPCR analysis of *cit D* |
| RT-3840-R | CGTCCTTTGATGTTGGGTTTC |  |
| RT-3842-F | CAAGGCAAAGTTGGTGGATTC | For RT-qPCR analysis of *cit E* |
| RT-3842-R | TCCCAGTTGGCACTCAAATAG |  |
