# Supplementary material for: Analysis of secondary metabolite gene clusters and chitin biosynthesis pathways of Monascus purpureus with high production of pigment and citrinin based on whole-genome sequencing
Source: PLoS One. 2022 Jun 1;17(6):e0263905. doi: 10.1371/journal.pone.0263905 (PMC9159588; doi:10.1371/journal.pone.0263905)
Supplement: Attachment — Submitted filename: Review_PlosOne_20220219.pdf [file pone.0263905.s002.pdf]

Analysis of secondary metabolite gene clusters and chitin biosynthesis pathways of *Monascus purpureus* with high production of pigment and citrinin based whole genome sequencing

In this study, the authors employed a hybrid sequencing strategy (PacBio and Illumina) to get the whole-genome assembly of a *Monascus purpureus* mutant. The authors comprehensively addressed the predicted secondary metabolite biosynthetic clusters as well as the expression of chitin and *Monascus* pigments genes in mutant strain, *Monascus purpureus* CSU M-183. Although there are some concerns (given below), I find this study relevant for the research community.

Comments:

1. In the Materials and methods section of WGS and assembling, the information about the method of PaBioRS II library preparation is not described. The authors should add information about the PacBio sequencing library construction and parameters used for filtering the reads. The authors should also provide information on the Illumina NGS sequencing platform used, and the processing of Illumina reads.

2. They used two sequencing platforms to generate the assembly of *Monascus purpureus* mutant. However, they did not describe how they validated the completeness of the genome assembly.

3. I am confused about the description of how they obtained the *M. purpureus* mutant strain. If they created the mutant strain from parent *M. purpureus* LQ-6, the authors have to describe the mutagenesis and screening. Otherwise, they have to cite the previous study that reported the *M. purpureus CSU*-M183 mutant.

4. Fig. 1 – I recommend giving the alphabets corresponding to each annotated function inside the pie chart. I find the pie chart shown in the manuscript not helpful because some colors are same for annotated functions. For example, the color for E, O, and Z are the same. The other option is providing the percentage of predicted genes with annotated function as a table instead of a pie chart.

5. In the methods section of RT-qPCR, the authors should add the statistical methods used for qPCR analysis. They did not mention how many replicates they used for qPCR experiments.

6. In the RT-qPCR results, the authors should give the p-values of significantly higher qPCR expression results instead of using terminologies such as “obviously higher”, “relatively significant”, “very significant,” or “extremely significant”. I find it hard to follow what the authors meant by “extremely significant” and “very significant”. They should also describe in the legend of Figure 4 on what each type of asterisks (**, ***) meant.

7. Page #14 (Line #18) - Page # 15 (Line #3) – "As shown in Fig. 4, phosphoacetylglucosamine mutase (PGM3) [EC:5.4.2.3] (encoded by gene g4907) converts N-acetyl-D-glucosamine 6-phosphate (GlcNAc-6P) to N-acetyl-alpha-D glucosamine 1-phosphate (GlcNAc-1P), which is then dephosphorylated by UDP-N22 acetylglucosamine diphosphorylase (UAP1) [EC:2.7.7.23] (encoded by gene g6630) to yield UDP-N-acetyl-D-glucosamine (UDP-GlcNAc)". The above description matches Fig 5, not Fig 4.

8. This manuscript requires language editing before accepting for publication. I have noticed the repetition of words, spelling, and grammatical errors. Although some paragraphs are fairly well written, I recommend revising the manuscript with the assistance of a native English speaker.

9. Authors should correct the format of in-text citation throughout the manuscript. Also, keep consistency in the units' format (Ex: ml or mL).
