# Supplementary material for: Analysis of secondary metabolite gene clusters and chitin biosynthesis pathways of Monascus purpureus with high production of pigment and citrinin based on whole-genome sequencing
Source: PLoS One. 2022 Jun 1;17(6):e0263905. doi: 10.1371/journal.pone.0263905 (PMC9159588; doi:10.1371/journal.pone.0263905)
Supplement: Attachment — Submitted filename: Response to Reviewers.docx [file pone.0263905.s003.docx]

ID: PONE-D-22-01622

Title: Analysis of secondary metabolite gene clusters and chitin biosynthesis pathways of *Monascus purpureus* with high production of pigment and citrinin based on whole-genome sequencing

Journal: Plos One

Dear Editor,

Thank you very much for your and reviewers' comments concerning our manuscript entitled “**Analysis of secondary metabolite gene clusters and chitin biosynthesis pathways of Monascus purpureus with high production of pigment and citrinin based on whole-genome sequencing**（PONE-D-22-01622）”. We have studied the editor/reviewer comments and made corrections that we hope meet with your approval. The main corrections have been marked in red in the revised manuscript (PONE-D-22-01622) and our details responses to the comments are given below in bold type.

**Editor' comments:**

When submitting your revision, we need you to address these additional requirements.

1. Please ensure that your manuscript meets PLOS ONE's style requirements, including those for file naming. The PLOS ONE style templates can be found at

https://journals.plos.org/plosone/s/file?id=wjVg/PLOSOne_formatting_sample_main_body.pdf and

https://journals.plos.org/plosone/s/file?id=ba62/PLOSOne_formatting_sample_title_authors_affiliations.pdf

**Response：Thank you very much for your comment,** **we have changed the manuscript to comply with PLOS ONE's style requirements, including file naming requirements**

2. Thank you for stating the following financial disclosure:

we would also like to thank Editage for English language editing. This work was supported by the National Natural Science Foundation of China (No. 32101906), Natural Science Foundation of Hunan Province (No. 2021JJ31146); Open Project Program of the Hunan Provincial Key Laboratory of Food Safety Monitoring and Early Waring (No. 2021KFJJ02), Education Department of Scientific Research Project of Hunan Province (No. 20B619) and Education Department of Postgraduate Research and Innovation Project of Hunan Province（No. CX20210863, CX20210898）

Please state what role the funders took in the study. If the funders had no role, please state: "The funders had no role in study design, data collection and analysis, decision to publish, or preparation of the manuscript."

If this statement is not correct you must amend it as needed.

**Response：Thank you for your comment. we have amended** **Role of Funder statement in the cover letter.**

3. Thank you for stating the following in the Acknowledgments Section of your manuscript:

Please note that funding information should not appear in the Acknowledgments section or other areas of your manuscript. We will only publish funding information present in the Funding Statement section of the online submission form.

Please remove any funding-related text from the manuscript and let us know how you would like to update your Funding Statement. Currently, your Funding Statement reads as follows:

we would also like to thank Editage for English language editing. This work was supported by the National Natural Science Foundation of China (No. 32101906), Natural Science Foundation of Hunan Province (No. 2021JJ31146); Open Project Program of the Hunan Provincial Key Laboratory of Food Safety Monitoring and Early Waring (No. 2021KFJJ02), Education Department of Scientific Research Project of Hunan Province (No. 20B619) and Education Department of Postgraduate Research and Innovation Project of Hunan Province（No. CX20210863, CX20210898）

Please include your amended statements within your cover letter; we will change the online submission form on your behalf.

**Response：Thank you for your comment, we have deleted the funding information in the manuscript and submitted them in the Funding Statement.**

4. We note that you have stated that you will provide repository information for your data at acceptance. Should your manuscript be accepted for publication, we will hold it until you provide the relevant accession numbers or DOIs necessary to access your data. If you wish to make changes to your Data Availability statement, please describe these changes in your cover letter and we will update your Data Availability statement to reflect the information you provide.

**Response：Thank you for your comment, we have provided accession numbers for the relevant data in the Materials and Methods of the manuscript (page 9, line 9-13).**

5. Your ethics statement should only appear in the Methods section of your manuscript. If your ethics statement is written in any section besides the Methods, please move it to the Methods section and delete it from any other section. Please ensure that your ethics statement is included in your manuscript, as the ethics statement entered into the online submission form will not be published alongside your manuscript.

**Response：Thank you for your comment, we have moved the ethics statement to the Materials and Methods section of the manuscript (page 9, line 14-16).**

6. Please include captions for your Supporting Information files at the end of your manuscript, and update any in-text citations to match accordingly. Please see our Supporting Information guidelines for more information: <http://journals.plos.org/plosone/s/supporting-information>.

**Response：Thank you for your comment, we have changed the supplemental information as requested (page 31, line 20-21).**

7. Please review your reference list to ensure that it is complete and correct. If you have cited papers that have been retracted, please include the rationale for doing so in the manuscript text, or remove these references and replace them with relevant current references. Any changes to the reference list should be mentioned in the rebuttal letter that accompanies your revised manuscript. If you need to cite a retracted article, indicate the article’s retracted status in the References list and also include a citation and full reference for the retraction notice.

**Response：Thank you for your comment,** **we have checked the reference list to make sure it is complete and correct.**

**Reviewers' comments:**

Reviewer #1:

The *Monascus purpureus* CSU-M183 strain is of industrial importance and warrants a detailed genomic analysis. The authors here have done a good job describing its genomic contents, particularly in relation to pigment and citrinin metabolism. I have a couple of comments.

1. Annotation pipelines such as MAKER pipeline take into account various evidences to predict the genes in a genome. It might perform better than solely using augustus and SNAP.

**Response：Thank you very much for your comment.**

2. In methods section, the genome assembly process is not described in enough details that it can be reproduced. Parameters used in HGAP program are missing.

**Response：Thank you very much for your comment, we have re-described the genome assembly process in detail in the Materials and methods section of WGS and assembling (page 7, line 4-7).**

3. In introduction, it is not clearly rationalized why the genome sequencing of this particular strain is necessary while several other high pigment producing strains are already sequenced.

**Response：Thank you very much for your comment. There are not many published whole genome sequences of *Monascus* so far, and there are certain differences between the *Monascus* species, such as some do not contain a complete monacolin K biosynthetic gene cluster, and some do not produce citrinin, etc. Besides, we illustrate that the cell wall is an ideal target for the regulation of fungal morphology, chitin is the main component of cell wall, but the number of genes encoding chitin synthase and chitin biosynthesis pathway in *M. purpureus* is still unclear (page 5, line 3-11). Therefore, we comprehensively predicted the gene encoding chitin synthase and the biosynthetic pathway of chitin in *M. purpureus* CSU-M183 through whole genome sequencing for future morphological studies.**

4.With several *M. purpureus* genomes available, it might be helpful to conduct a comparative genomic analysis among them to elucidate likely mechanism that CSU-M183 strain is special in its metabolic capacity.

**Response：Thank you very much for your suggestion. Based on the whole genome sequence of strain CSU-M183, we have performed genome resequencing of the mutant strains (morphologically different strain), focusing on the genes related to morphology, metabolism and growth, and to explore the relationship between the morphology, growth and metabolism of *Monascus*, for our future study.**

Reviewer #2:

In this study, the authors employed a hybrid sequencing strategy (PacBio and Illumina) to get the whole-genome assembly of a *Monascus purpureus* mutant. The authors comprehensively addressed the predicted secondary metabolite biosynthetic clusters as well as the expression of chitin and *Monascus* pigments genes in mutant strain, *Monascus purpureus* CSU-M183. Although there are some concerns (given below), I find this study relevant for the research community.

Comments:

1. In the Materials and methods section of WGS and assembling, the information about the method of PaBioRS II library preparation is not described. The authors should add information about the PacBio sequencing library construction and parameters used for filtering the reads. The authors should also provide information on the Illumina NGS sequencing platform used, and the processing of Illumina reads.

**Response：Thank you very much for you comment,** **we have added relavant information in the Materials and methods section of WGS and assembling (page 7 line 8 to page 8 line 6).**

2. They used two sequencing platforms to generate the assembly of *Monascus purpureus* mutant. However, they did not describe how they validated the completeness of the genome assembly.

**Response：Thank you very much for you comment. Indeed the genome assembly was performed using HGAP2 for PacBio RSII reads, and Illumina reads were mapped on assembled genome to correct possible SNPs and Indels. Though we finally obtained a high-quality genome with 69 contigs, it’s not assembled into chromosome level. So we did not estimate the completeness of the genome (page 7 line 8 to page 8 line 6).**

3. I am confused about the description of how they obtained the *M. purpureus* mutant strain. If they created the mutant strain from parent *M. purpureus* LQ-6, the authors have to describe the mutagenesis and screening. Otherwise, they have to cite the previous study that reported the *M. purpureus* CSU-M183 mutant.

**Response：Thank you very much for you comment, we have cited our previous research of the *M. purpureus* CSU-M183 mutant**（**page 6, line 2）.**

4. Fig. 1 – I recommend giving the alphabets corresponding to each annotated function inside the pie chart. I find the pie chart shown in the manuscript not helpful because some colors are same for annotated functions. For example, the color for E, O, and Z are the same. The other option is providing the percentage of predicted genes with annotated function as a table instead of a pie chart.

**Response：Thank you very much for you comment, the percentage of predicted genes with annotation functions have been presented in a table (Table 2).**

5. In the methods section of RT-qPCR, the authors should add the statistical methods used for qPCR analysis. They did not mention how many replicates they used for qPCR experiments.
**Response：Thank you very much for you comment. We have supplemented Statistical analysis in the Materials and Methods to illustrate the statistical methods used in this study and the number of experimental replicates (page 9, line 17-22).**

6. In the RT-qPCR results, the authors should give the p-values of significantly higher qPCR expression results instead of using terminologies such as “obviously higher”, “relatively significant”, “very significant,” or “extremely significant”. I find it hard to follow what the authors meant by “extremely significant” and “very significant”. They should also describe in the legend of Figure 4 on what each type of asterisks (**, ***) meant.

**Response：Thank you very much for your comment, we gave the p-values of significantly higher qPCR expression results and supplemented in the legend of Fig 3 on what each type of asterisks meant (page 22, line 12-13).**

7. Page #14 (Line #18) - Page # 15 (Line #3) – “As shown in Fig. 4, phosphoacetylglucosamine mutase (PGM3) [EC:5.4.2.3] (encoded by gene g4907) converts N-acetyl-D-glucosamine 6-phosphate (GlcNAc-6P) to N-acetyl-alpha-D glucosamine 1-phosphate (GlcNAc-1P), which is then dephosphorylated by UDP-N22 acetylglucosamine diphosphorylase (UAP1) [EC:2.7.7.23] (encoded by gene g6630) to yield UDP-N-acetyl-D-glucosamine (UDP-GlcNAc)”. The above description matches Fig 5, not Fig 4.

**Response：Thank you very much for your comment，we have corrected it.**

8. This manuscript requires language editing before accepting for publication. I have noticed the repetition of words, spelling, and grammatical errors. Although some paragraphs are fairly well written, I recommend revising the manuscript with the assistance of a native English speaker.
**Response：Thank you for your comment, the manuscript has been revised with the help of native English speakers.**

9. Authors should correct the format of in-text citation throughout the manuscript. Also, keep consistency in the units' format (Ex: ml or mL)

**Response：Thank you very much for your comment, we have changed the in-text citation format as requested and unified the unit of volume to ml.**
