# Supplementary material for: Analysis of secondary metabolite gene clusters and chitin biosynthesis pathways of Monascus purpureus with high production of pigment and citrinin based on whole-genome sequencing
Source: PLoS One. 2022 Jun 1;17(6):e0263905. doi: 10.1371/journal.pone.0263905 (PMC9159588; doi:10.1371/journal.pone.0263905)
Supplement: Attachment — Submitted filename: Response to Reviewers.docx [file pone.0263905.s004.docx]

ID: PONE-D-22-01622R2

Title: Analysis of secondary metabolite gene clusters and chitin biosynthesis pathways of *Monascus purpureus* with high production of pigment and citrinin based on whole-genome sequencing

Journal: Plos One

Dear Editor,

Thank you very much for your and reviewers' comments concerning our manuscript entitled “**Analysis of secondary metabolite gene clusters and chitin biosynthesis pathways of *Monascus purpureus* with high production of pigment and citrinin based on whole-genome sequencing**（PONE-D-22-01622R2）”. We have studied the editor/reviewer comments and made corrections that we hope meet with your approval. Major corrections in the revised manuscript (PONE-D-22-01622R2) have been highlighted (blue) and our details responses to the comments are given below in bold type.

**Editor' comments:**

1. Please submit the PacBio sequencing reads (raw data) and Illumina data you generated to an appropriate database (e.g. NCBI SRA) and give the SRA accession number. Currently, it appears as if you only submitted the assembled genome, but not the raw sequence data, which are also important.

**Response：Thank you very much for your comment. Firstly, due to the COVID-19 in Shanghai, the people were strongly asked to work at home, and the raw date is being stored on the server of sequencing company (Chinese National Human Genome Center at Shanghai). Secondly, we would submit the raw date to NCBI-SRA as soon as possible when the staff return to work. Thus, we just provided the raw date of SRA acceptance number with BioProject of PRJNA824977 in this revised manuscript** (**PONE-D-22-01622R2；Page 9，Line 14-15).**

2. Some sections of the manuscript were apparently changed in an attempt to improve the English, but this is not actually better in many cases. Especially the abstract now contains a number of language problems in the changes sentences that were not present before. Please carefully check the manuscript and correct language problems.

**Response：Thank you for your comment, the manuscript has been revised and marked in blue.**

**Reviewers' comments:**

Reviewer #1:

1. Reviewer 1 asked about the reasons why this particular *Monascus purpureus* strain was sequenced. This is still not sufficiently explained in the introduction. Please explain what makes this strain interesting (not the species as such, but this particular strain).

**Response：Thank you very much for your comment. We have explained what makes this strain interesting in the introduction（Page 5, Line 6-16）.**

Reviewer #2:

Reviewer 2 suggested checking for genome completeness, which is something that can and should be done especially with genomes that are not assembled fully at chromosome level. You can use the presence of conserved genes to check for completeness (of the gene space at least), e.g. by using the BUSCO tool or other methods to check for the presence of expected conserved eukaryotic genes. Please do this and document the method used and the results in your manuscript.

Comments:

**Response：Thank you very much for you comment. We have checked the completeness of the genome by using the BUSCO** **tool. The corresponding methods（Page 6, Line 20-22） and results（Page 10, Line 9-13）have been supplemented in the Manuscript.**
